# Disproportionality analysis of quinolone safety in children using data from the FDA adverse event reporting system (FAERS)

**DOI:** 10.3389/fped.2022.1069504

**Published:** 2023-01-11

**Authors:** Wenqiang Kong, Wei Mao, Lin Zhang, Yanyan Wu

**Affiliations:** ^1^ Department of Pharmacy, Zigong First People’s Hospital, Zigong, China; ^2^ Department of Pharmacy, Nanan People’s Hospital of Chongqing, Chongqing, China; ^3^ Department of Pharmacy, Southwest Hospital of Army Medical University (Third Military Medical University), Chongqing, China; ^4^ Department of Pharmacy, Women and Children’s Hospital of Chongqing Medical University, Chongqing, China; ^5^ Department of Pharmacy, Chongqing Health Center for Women and Children, Chongqing, China

**Keywords:** drug safety, children, quinolones, pharmacovigilance, disproportionality analysis

## Abstract

**Background:**

Quinolones are widely prescribed for the treatment or prevention of infectious diseases in children. To gain further insight into quinolone-associated adverse event (AE) in children and better protect pediatric patients, continued surveillance of safety data is essential. The purpose of this study was to characterize the safety profiles of quinolone-associated AEs in children by mining the FDA adverse event reporting system (FAERS).

**Methods:**

FAERS reports from quarter 1 of 2004 to quarter 1 of 2022 were included in the study. The Medical Dictionary for Regulatory Activities (MedDRA) was used to identify adverse events. Reporting odds ratios (ROR) corresponding 95% confidence intervals (CIs) and information component (IC) along with 95% CIs were calculated to detect drug–AE pairs with higher-than-expected reporting rates within the FAERS from System Organ Classes (SOCs) to Preferred Terms (PTs). Reports were considered as signals if the 95% conﬁdence interval did not contain the null value.

**Results:**

After inclusion criteria were applied, a total of 4,704 reports associated with quinolones were considered. Most FAERS reports associated with ciprofloxacin (*N* = 2,706) followed by levofloxacin (*N* = 1,191), moxifloxacin (*N* = 375), oflaxacin (*N* = 245) and ozenoxacin (*N* = 187). The most common age group was 12–18 years. The median weight was 39.0 kilogram. The adverse effects of quinolones emerging for SOCs primarily included Infections and infestations, gastrointestinal symptoms, blood and lymphatic system disorders, cardiac disorders, nervous system disorders, musculoskeletal and connective tissue disorders and psychiatric disorders. The most frequently AE signals at the PT level were pyrexia (*N* = 236), febrile neutropenia (*N* = 120), off label use (*N* = 48), drug resistance (*N* = 18) and cardiac arrest (*N* = 22) following the use of ciprofloxacin, levofloxacin, moxifloxacin, ofloxacin, and ozenoxacin, respectively. Serious oznoxacin-associated AE signals were found and have not been documented in the package insert. They included cardiac arrest (*N* = 22; ROR = 19.83; IC = 3.68), overdose (*N* = 21; ROR = 4.98; IC = 2.07), seizure (*N* = 16; ROR = 6.01; IC = 2.29), small for dates baby (*N* = 9; ROR = 14.7; IC = 3.05), completed suicide (*N* = 15, ROR = 18.87; IC = 3.51), asthma (*N* = 9; ROR = 6.69; IC = 2.24;) and hypotension (*N* = 9; ROR = 3.83; IC = 1.68).

**Conclusion:**

This study provided additional evidence with respect to quinolones-related AEs for children. Generally, the findings of this study are compatible with AEs recorded in package inserts. The unexpected signals of ozenoxacin justify active vigilance by clinicians and timely monitoring by pharmacovigilance experts.

## Introduction

Nalidixic acid was accidentally discovered as a chloroquine byproduct in 1962 and several additional quinolones, including ciproﬂoxacin, levoﬂoxacin, and moxiﬂoxacin, have since been marketed. These are widely prescribed for the treatment of infectious diseases in both inpatient and outpatient adults because of their broad-spectrum antimicrobial activity, excellent oral absorption, high tissue penetration, and long elimination half-life which permits once-daily dosing (1). While fluoroquinolones (FQs) also have favorable pharmacokinetic properties in children, the response to these drugs differs between children and adults (2). In addition, the efficacy of FQs in children is similar to other antibiotics for the treatment of respiratory infections, cystic fibrosis, febrile neutropenia, and urinary tract infections (3–7). Some FQs have FDA approval for the treatment of complicated urinary tract infections, inhalation anthrax, and pyelonephritis in children (8). As the prevalence of antibiotic-resistant pathogens such as *Pseudomonas aeruginosa*, *Tuberculosis*, and *Mycoplasma*, has increased, FQs are being increasingly prescribed to children. In the United States, 372,357 oral FQs were dispensed to children from 2006 to 2016 (9).

Quinolone safety is an area of focus in pediatrics. Early toxicology studies in juvenile animals found that quinolones can induce dose-related arthrotoxicity (10, 11), leading to a ban on their use among children aged <18 years in China. In addition, a clinical trial about the safety of ciprofloxacin in children demonstrated that the ciprofloxacin group had a small but statistically significant increase in musculoskeletal adverse events (AEs) in comparison to the control group (9.3% and 6%, respectively) 6 weeks after treatment (8). Another randomized controlled study of pediatric patients with complicated intra-abdominal infections found that QT prolongation, a heart rhythm disorder, was higher in the moxifloxacin group than in the control group (7% and 1.3%, respectively) (2). However, other studies found no AEs associated with FQ use in children (8). Human studies have shown that there is a very low risk of FQ-associated musculoskeletal disorders. A 5-year follow-up study indicated that levofloxacin-treated children reported slightly lower musculoskeletal AEs than comparator children, and indicated that the risk of cartilage injury was rare (12). Moreover, no growth abnormalities were associated with FQ treatment in children (12). In 2018, a multicenter study (NCT01069900) examined the efficacy and safety of moxifloxacin in children with complicated intra-abdominal infections (2). The study found that after 5–14 days of treatment, the overall incidence of AEs was similar between the moxifloxacin and comparator groups and most AEs were mild or moderate. The proportion of children who experienced musculoskeletal AEs was comparable between the two treatment arms (moxifloxacin group: 4.3%; comparator group: 3.3%) (2). A recent systematic review including 154,638 children found that the most common AEs associated with systemic quinolones were gastrointestinal reactions such as diarrhea, vomiting, nausea, and abdominal pain (13). This study also found that the quinolone-treated children had a higher risk of musculoskeletal AEs than those in the comparator group after 2–12 months of follow-up (2–6 months: RR 2.56, 95% CI, 2.26–2.89; 7–12 months: RR 1.35, 95% CI, 0.98–1.86); however, the incidence of musculoskeletal AEs was uncommon and reversible (13). Considering the inadequacy of long-term safety studies, the American Academy of Pediatrics (AAP) has recommended FQ use in children only when no effective and safe alternative is available (7).

While a few studies have identified other AEs associated with quinolone use among pediatric patients, such as cardiotoxicity and neurotoxicity, most have focused on musculoskeletal side effects. In addition, except for levofloxacin, few large and long-term studies of FQ safety have been conducted in children. To gain further insight into quinolone-associated AEs in children and better protect pediatric patients, continued surveillance of safety data is essential. Data mining is a useful method for monitoring the safety of new drugs and regularly supervising the effects of established drugs. To our knowledge, no studies have mined the FDA adverse events reporting system (FAERS) database for AEs and adverse outcomes associated with the clinical use of systemic or topical quinolones in children. To address this, the current study sought to characterize and map the safety profiles of quinolone-associated AEs in children using FAERS data.

## Materials and methods

### Data source

FAERS is a publicly available database that collects AE and medication error-associated reports submitted by healthcare professionals, consumers, and drug manufactures (14). FAERS contains information on patient demographics, drug characteristics, and AEs and is updated quarterly by the FDA. Data for this study, including patient baseline information, reporter, report type, report region, drug role, AE outcomes, indications, therapy duration, and route of administration, were extracted from the FAERS database.

### Study design

Full data from quarter 1 of 2004 (2004Q1) to quarter 1 of 2022 (2022Q1) were downloaded from the FAERS website in ASCII format (15). AE reports associated with quinolone treatment of children <18 years of age were included. Prior to statistical analysis, duplicate reports were identified and removed according to the FDA’s recommendation for adopting the most recent case number (16).

### Definition of drug exposure

Reports involving systemic or topical quinolone use were extracted. Quinolones, including besiﬂoxacin, ciproﬂoxacin, gatiﬂoxacin, gemiﬂoxacin, grepaﬂoxacin, levoﬂoxacin, lomeﬂoxacin, moxiﬂoxacin, norﬂoxacin, oﬂoxacin, sparﬂoxacin, temaﬂoxacin, trovaﬂoxacin, and ozenoxacin, were identified by searching for their generic and brand names in the Drugs@FDA database (17). Generic names, brand names, and abbreviations were used to distinguish between quinolone-related records in the DRUG files. More than 100 individual reports of quinolone use were included.

### Definition of adverse events

AEs were coded in the FAERS using Preferred Terms (PT) derived from the Medical Dictionary for Regulatory Activities (MedDRA®) (https://www.medalerts.org/vaersdb/meddra/). Each PT was given one or more high-level term (HLT), high-level group term (HLGT), and system organ class (SOC) level in MedDRA (18). Serious AEs were defined as any quinolone-associated AEs associated with death, a life-threatening outcome, initial or prolonged hospitalization, disability, congenital anomaly, required intervention, or another important medical event (19). Cases defined as AEs reports in which the reporter mentioned quinolones as suspect (“Primary Suspect”, “Secondary Suspect”, or “Concomitant”) were included for disproportional analysis.

### Statistical analysis

Two quantitative disproportionality methods, the Bayesian and frequentist methods, were used to detect AEs (see formula in the Supplementary material). First, reporting odds ratios (ROR), which correspond to the lower limit of 95% confidence intervals (ROR_025_), were calculated to define a potential association between AEs and individual quinolone use. An ROR_025_ > 1 and the existence of at least three cases was defined as a significant signal (20). A higher ROR indicated a stronger association between quinolones and AEs. While the ROR algorithm is clear and easy to understand, it has a tendency to find false-positive results when a small number of reports is included (21). Thus, we calculated the information component (IC) along with the lower limit of 95% confidence intervals (IC_025_) using the Bayesian confidence propagation network (BCPNN) algorithm. This protects from over-sensitivity to extremely low expected cases and low observed cases (20, 22). An IC_025_ of >0 is defined as a signal (20). Descriptive analysis was used to obtain the results. Frequencies and percentages were used for dichotomous variables. Median and interquartile ranges were used for continuous variables. Data management and analysis were performed using PostgreSQL (version 14.4) and R software (version 4.2.0), respectively.

## Results

### Baseline characteristics of children receiving quinolones

The baseline characteristics of the pediatric study population are shown in Table 1. Between 1Q 2004 and 1Q 2022, 14,796,988 AE reports, of which 488,969 involved children, were submitted to FAERS. Of these, 4,704 reports met the inclusion criteria, and 484,265 reports were excluded because quinolones were not be mentioned. A total of 2,706 AE reports were submitted for ciprofloxacin, 1,191 for levofloxacin, 375 for moxifloxacin, 245 for ofloxacin, and 187 for ozenoxacin. There was a slightly higher proportion of reports among female than male children (49.7% and 47.6%, respectively), the median age of the study population was 11 years, and the median weight was 39.0 kilogram. The most common age group was 12–18 years (47.4%). About 80% of the reports were expedited and most were submitted by a health professional (39.3%) or physician (29.2%). More than half (54.3%) of the reports considered quinolones to be a concomitant drug in the occurrence of AEs, 28.4% considered quinolones to be the primary suspect drug, and 16.7% of AE listed quinolones as the suspected secondary exposure. The most common drugs accompanied by quinolones were methotrexate (*n* = 409), followed by meropenem (*n* = 378), vancomycin (*n* = 374), and ondansetron (*n* = 367) (see Supplementary Figure S1). The most reported outcomes were initial or prolonged hospitalization (38.3%), followed by other serious outcomes (28.8%), and death (11.4%). A total of 41.9% of the events were reported from the United States. Most AE reports in children (74.5%) did not indicate the route of administration. Of those with a known route, 8.2% of quinolones were administered orally, and 5.6% were given intravenously. Ozenoxacin was topically administered to children.

**Table 1:** The baseline characteristics of adverse events reports related to quinolones for children.

Characteristics	Ciprofloxacin (*N* = 2706)	Levofloxacin (*N* = 1191)	Moxifloxacin (*N* = 375)	Ofloxacin (*N* = 245)	Ozenoxacin (*N* = 187)	Overall (*N* = 4704)
Age group, (y)						
Median (Q1, Q3)	11.0 (5.00, 15.0)	11.0 (6.00, 15.0)	10.0 (5.00, 14.0)	8.00 (3.00, 14.0)	13.0 (5.00, 16.0)	11.0 (5.00, 15.0)
0–3	444 (16.4%)	181 (15.2%)	65 (17.3%)	55 (22.4%)	45 (24.1%)	790 (16.8%)
3–6	294 (10.9%)	112 (9.4%)	50 (13.3%)	49 (20.0%)	5 (2.7%)	510 (10.8%)
6–12	672 (24.8%)	323 (27.1%)	89 (23.7%)	57 (23.3%)	32 (17.1%)	1173 (24.9%)
12–18	1296 (47.9%)	575 (48.3%)	171 (45.6%)	84 (34.3%)	105 (56.1%)	2231 (47.4%)
Children sex						
Female	1334 (49.3%)	596 (50.0%)	204 (54.4%)	110 (44.9%)	96 (51.3%)	2340 (49.7%)
Male	1297 (47.9%)	573 (48.1%)	157 (41.9%)	129 (52.7%)	85 (45.5%)	2241 (47.6%)
Unknown	75 (2.8%)	22 (1.8%)	14 (3.7%)	6 (2.4%)	6 (3.2%)	123 (2.6%)
Weight, (Kg)						
Median (Q1, Q3)	42.0 (20.0, 58.0)	42.0 (21.1, 58.0)	25.6 (15.0, 50.0)	25.1 (14.0, 47.6)	28.3 (2.99, 59.6)	39.0 (19.0, 57.7)
Unknown	1577 (58.3%)	634 (53.2%)	223 (59.5%)	149 (60.8%)	119 (63.6%)	2702 (57.4%)
Report type						
30-Day	0 (0%)	0 (0%)	0 (0%)	1 (0.4%)	0 (0%)	1 (0.0%)
Direct	194 (7.2%)	113 (9.5%)	29 (7.7%)	12 (4.9%)	8 (4.3%)	356 (7.6%)
Expedited (15-Day)	2206 (81.5%)	950 (79.8%)	283 (75.5%)	184 (75.1%)	141 (75.4%)	3764 (80.0%)
Periodic (Non-Expedited)	306 (11.3%)	128 (10.7%)	63 (16.8%)	48 (19.6%)	38 (20.3%)	583 (12.4%)
Reporter						
Physician	811 (30.0%)	363 (30.5%)	94 (25.1%)	63 (25.7%)	41 (21.9%)	1372 (29.2%)
Pharmacist	183 (6.8%)	82 (6.9%)	22 (5.9%)	23 (9.4%)	15 (8.0%)	325 (6.9%)
Other health-professional	1030 (38.1%)	473 (39.7%)	169 (45.1%)	95 (38.8%)	83 (44.4%)	1850 (39.3%)
Lawyer	16 (0.6%)	5 (0.4%)	0 (0%)	1 (0.4%)	2 (1.1%)	24 (0.5%)
Consumer	488 (18.0%)	214 (18.0%)	63 (16.8%)	39 (15.9%)	37 (19.8%)	841 (17.9%)
Unknown	178 (6.6%)	54 (4.5%)	27 (7.2%)	24 (9.8%)	9 (4.8%)	292 (6.2%)
Country of the reporters						
United States	1030 (38.1%)	597 (50.1%)	124 (33.1%)	107 (43.7%)	113 (60.4%)	1971 (41.9%)
United Kingdom	271 (10.0%)	20 (1.7%)	43 (11.5%)	12 (4.9%)	8 (4.3%)	354 (7.5%)
Canada	184 (6.8%)	126 (10.6%)	23 (6.1%)	1 (0.4%)	9 (4.8%)	343 (7.3%)
Italy	180 (6.7%)	53 (4.5%)	20 (5.3%)	0 (0%)	0 (0%)	253 (5.4%)
France	139 (5.1%)	39 (3.3%)	20 (5.3%)	19 (7.8%)	2 (1.1%)	219 (4.7%)
Japan	39 (1.4%)	68 (5.7%)	6 (1.6%)	27 (11.0%)	0 (0%)	140 (3.0%)
India	37 (1.4%)	38 (3.2%)	28 (7.5%)	26 (10.6%)	0 (0%)	129 (2.7%)
Germany	72 (2.7%)	27 (2.3%)	6 (1.6%)	4 (1.6%)	35 (18.7%)	144 (3.1%)
Other Country	663 (24.5%)	207 (17.4%)	99 (26.4%)	39 (15.9%)	14 (7.5%)	1022 (21.7%)
Unknown	91 (3.4%)	16 (1.3%)	6 (1.6%)	10 (4.1%)	6 (3.2%)	129 (2.7%)
Drug role in AEs						
Primary suspect	760 (28.1%)	326 (27.4%)	142 (37.9%)	57 (23.3%)	53 (28.3%)	1338 (28.4%)
Secondary suspect	379 (14.0%)	195 (16.4%)	85 (22.7%)	74 (30.2%)	51 (27.3%)	784 (16.7%)
Concomitant	1544 (57.1%)	670 (56.3%)	143 (38.1%)	113 (46.1%)	83 (44.4%)	2553 (54.3%)
Interacting	23 (0.9%)	0 (0%)	5 (1.3%)	1 (0.4%)	0 (0%)	29 (0.6%)
Outcome of AEs						
Death	347 (12.8%)	122 (10.2%)	26 (6.9%)	19 (7.8%)	22 (11.8%)	536 (11.4%)
Life-Threatening	199 (7.4%)	140 (11.8%)	22 (5.9%)	12 (4.9%)	22 (11.8%)	395 (8.4%)
Hospitalization-Initial or Prolonged	1055 (39.0%)	515 (43.2%)	96 (25.6%)	89 (36.3%)	47 (25.1%)	1802 (38.3%)
Disability	59 (2.2%)	37 (3.1%)	7 (1.9%)	11 (4.5%)	2 (1.1%)	116 (2.5%)
Congenital Anomaly	22 (0.8%)	2 (0.2%)	0 (0%)	1 (0.4%)	8 (4.3%)	33 (0.7%)
Required Intervention to Prevent Permanent Impairment/Damage	10 (0.4%)	11 (0.9%)	2 (0.5%)	0 (0%)	0 (0%)	23 (0.5%)
Important Medical Event	763 (28.2%)	300 (25.2%)	165 (44.0%)	71 (29.0%)	55 (29.4%)	1354 (28.8%)
Unknown	251 (9.3%)	64 (5.4%)	57 (15.2%)	42 (17.1%)	31 (16.6%)	445 (9.5%)
Route of administration						
Intravenous	172 (6.4%)	66 (5.5%)	24 (6.4%)	1 (0.4%)	0 (0%)	263 (5.6%)
Ophthalmic	15 (0.6%)	1 (0.1%)	65 (17.3%)	23 (9.4%)	0 (0%)	104 (2.2%)
Oral	255 (9.4%)	84 (7.1%)	27 (7.2%)	19 (7.8%)	0 (0%)	385 (8.2%)
Otic	130 (4.8%)	0 (0%)	0 (0%)	11 (4.5%)	0 (0%)	141 (3.0%)
Topical	9 (0.3%)	2 (0.2%)	3 (0.8%)	7 (2.9%)	187 (100%)	208 (4.4%)
Other	30 (1.1%)	8 (0.7%)	4 (1.1%)	2 (0.8%)	0 (0%)	44 (0.9%)
Unknown	2095 (77.4%)	1030 (86.5%)	252 (67.2%)	182 (74.3%)	0 (0%)	3559 (75.7%)

### Fluoroquinolone indications

The indications of the top 50 most frequently reported quinolone-associated AEs are shown in Supplementary Figure S2. Of these, the most common quinolone-associated indication was prophylaxis (*n* = 369), followed by tuberculosis (*n* = 272), urinary tract infection (*n* = 116), and *Pseudomonas* infection (*n* = 64). Among individual antibiotics, ciprofloxacin was frequently prescribed for prophylaxis (*n* = 243), urinary tract infection (*n* = 100), pseudomonas infection (*n* = 54), and Crohn’s disease (*n* = 40), levofloxacin was frequently administered for prophylaxis (*n* = 114), tuberculosis (*n* = 108), infection prophylaxis (*n* = 32), and sinusitis (*n* = 30), moxifloxacin was frequently used for tuberculosis (*n* = 105), conjunctivitis (*n* = 20), sinusitis (*n* = 12), and *Mycobacterium abscessus* infection (*n* = 10), ofloxacin was commonly given for tuberculosis (*n* = 44), and ozenoxacin was frequently prescribed for sleep disorder (*n* = 12) and abnormal behaviour (*n* = 11).

### Quinolone treatment duration

The duration of quinolone treatment is calculated as the time from when the drug therapy was initiated to the time drug use was stopped. Since the start date of drug use was set as day 1, an additional day was added to the treatment time. The upper limit of therapy duration was set at 365 days. Median treatment time was 7 days for ciprofloxacin, 8 days for levofloxacin, 5 days for moxifloxacin and ofloxacin, and 41 days for ozenoxacin.

### Ae signals associated with quinolones

**Figure 1:**
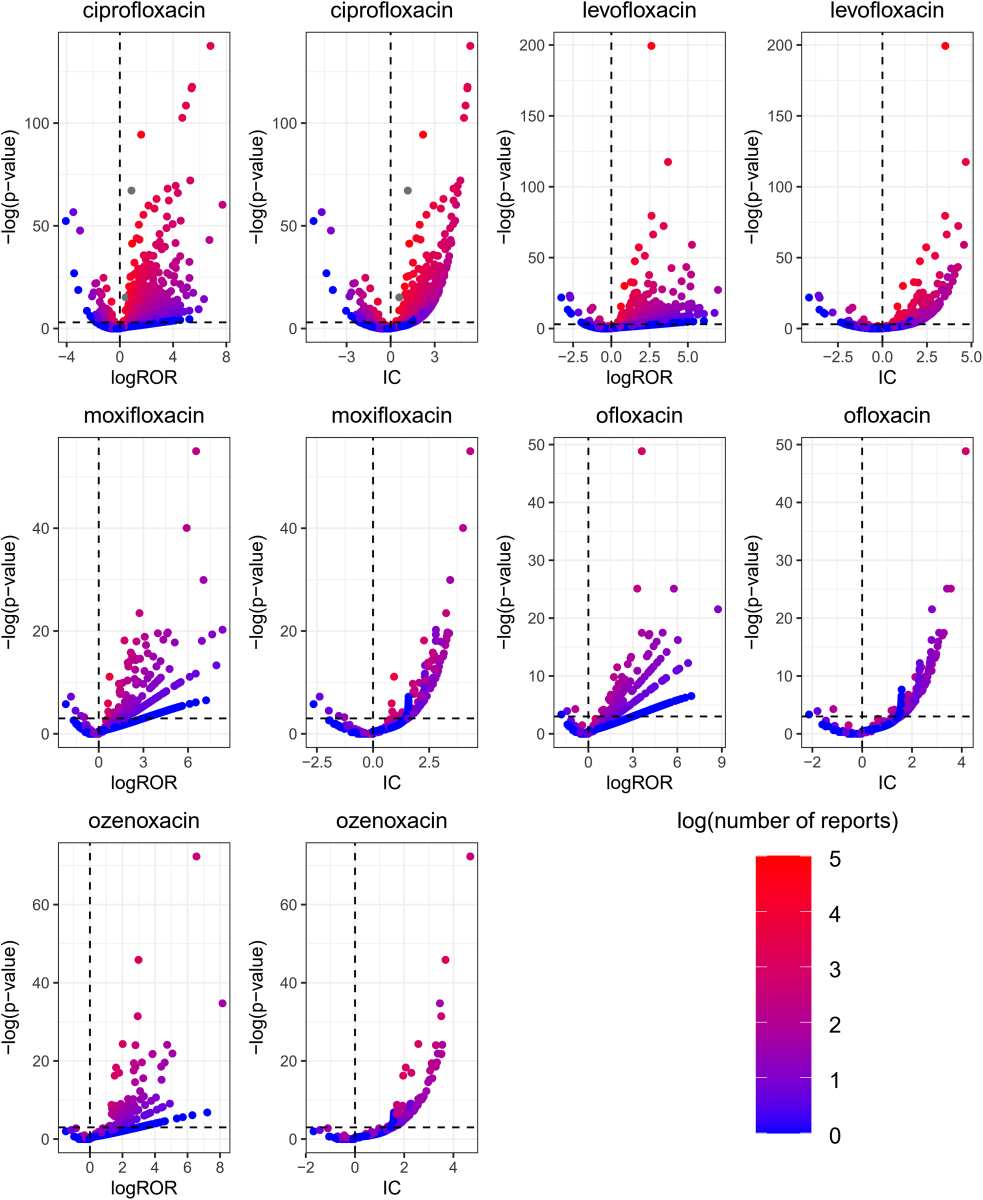
Quinolones associated with signals of AE reports. The x-axis is the logarithm of the reporting ROR (log ROR) or IC, and the Y-axis is the negative logarithm of the *P*-value calculated using Fisher’s exact test (− log *P*-value). The positive y-direction represents a strongly significant difference. The colors of the individual points represent differences in the log of the number of reports for each PT. In this scatterplot, the signal is larger for the points plotted in the upper right corner. The blue-to-red colors represent the number of times an adverse effect was reported.

**Figure 2:**
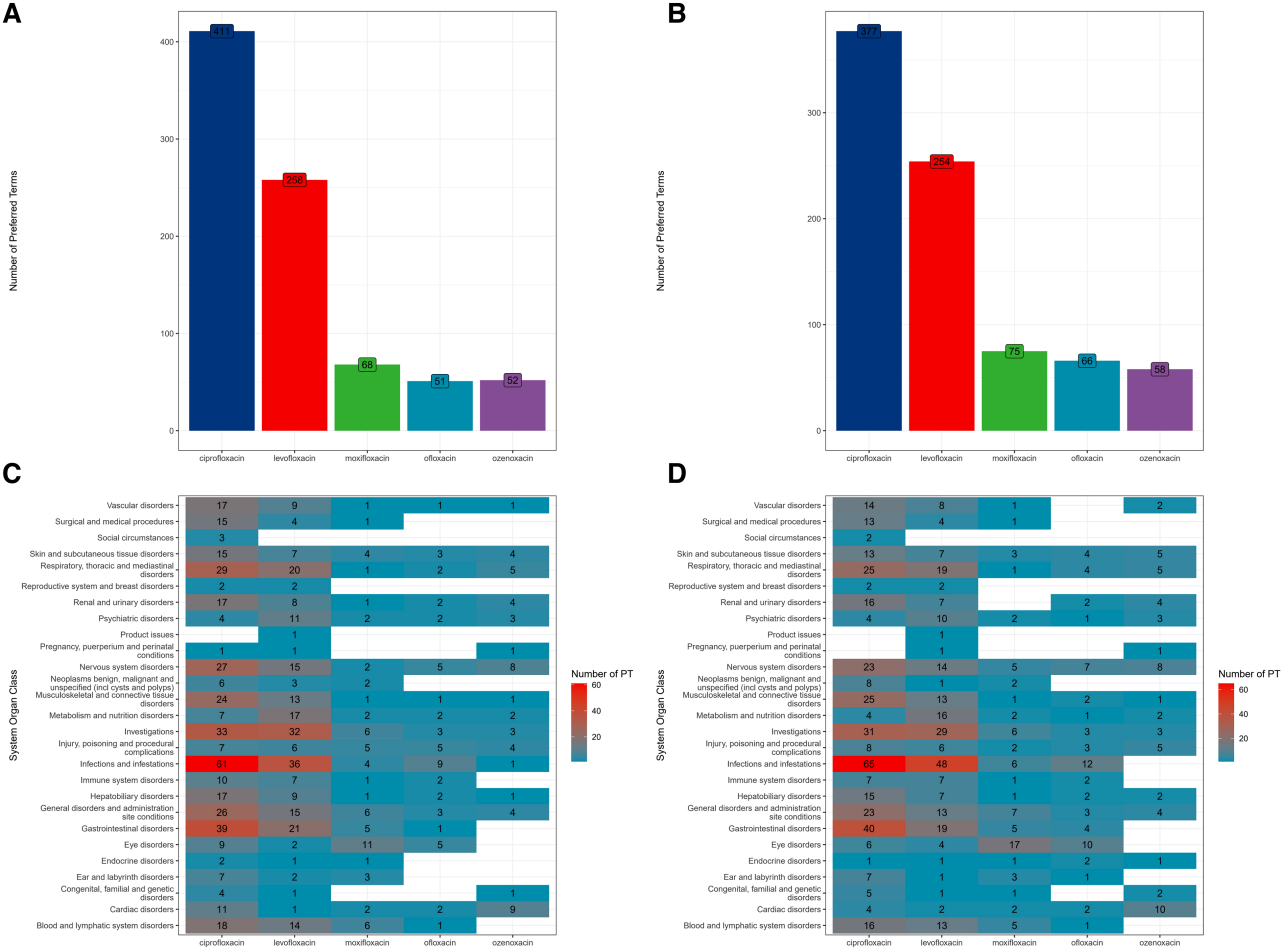
The number of signals of AE reports at SOC and PT levels using ROR and BCPNN algorithms. (**A**) signals of AE reports detected by ROR algorithm; (**B**) signals of AE reports detected by BCPNN algorithm; (**C**) signals of AE reports detected by ROR algorithm at SOC level; (**D**) signals of AE reports detected by BCPNN algorithm at SOC level.

**Figure 3:**
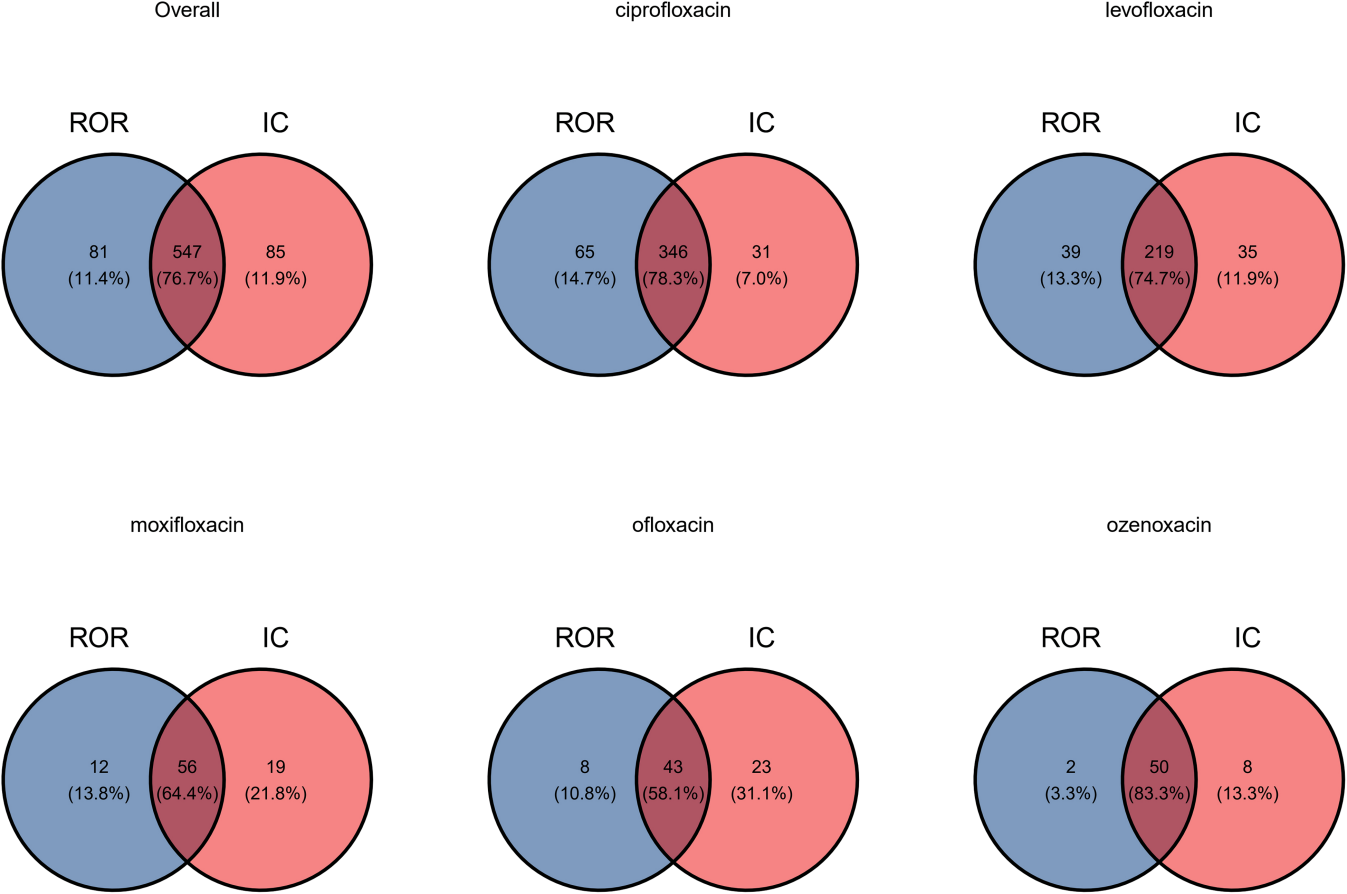
The Venn diagram for signals of AE reports of quinolones.

A volcano plot was developed to investigate the relationship between the ROR, IC, and significant differences to identify quinolones and other drugs that were associated with AEs (Figure 1) (23). Both the signal and the significant differences for AEs plotted in the upper right side of the figure were larger than they were for other drugs. ROR and BCPNN algorithms were used to detect the AE signals at the system organ class (SOC) and PT level. AE signals associated with the use of quinolones in children are shown in Figure 2. The two algorithms found similar numbers of AE signals for this drug class (see Figure 3). Ciprofloxacin was associated with the highest number of signals, followed by levofloxacin, moxifloxacin, ofloxacin, and ozenoxacin. Considering quinolones as a class, the top ten most commonly observed AE signals using the BCPNN/ROR algorithm in the following SOC included infections and infestations (*n* = 131/111), investigations (*n* = 72/77), gastrointestinal disorders (*n* = 68/66), nervous system disorders (*n* = 57/57), respiratory, thoracic and mediastinal disorders (*n* = 54/57), general disorders and administration site conditions (*n* = 50/54), musculoskeletal and connective tissue disorders (*n* = 42/40), blood and lymphatic system disorders (*n* = 39/35), and skin and subcutaneous tissue disorders (*n* = 33/35) The top 20 most frequently reported AE signals at the PT level are shown in Table 2. Pyrexia [*n* = 236; ROR = 2.39; 95% CI (2.09–2.74); IC = 1.17; 95% CI (0.95–1.33)], febrile neutropenia [*n* = 120; ROR = 13.74; 95% CI (11.35–16.64); IC = 3.54; 95% CI (3.23–3.75)], off label use [*n* = 48; ROR = 2.07; 95% CI (1.53–2.81); IC = 0.94; 95% CI (0.46–1.28)], drug resistance [*n* = 18; ROR = 36.74; 95% CI (22.65–59.59); IC = 4.16; 95% CI (3.37–4.71)], and cardiac arrest [*n* = 22; ROR = 19.83; 95% CI (12.69–30.98); IC = 3.68; 95% CI (2.96–4.18)] were associated with the highest number of reports following the use of ciprofloxacin, levofloxacin, moxifloxacin, ofloxacin, and ozenoxacin, respectively. Levofloxacin and ciprofloxacin were also associated with musculoskeletal AEs such as arthralgia [levofloxacin: *n* = 58; ROR = 6.02; 95% CI (4.62–7.58); IC = 2.46; 95% CI (2.02–2.77); ciprofloxacin: *n* = 51; ROR = 2.25; 95% CI (1.7–2.97); IC = 1.13; 95% CI (0.66–1.66)]. Cardiac arrest [*n* = 10; ROR = 4.06; 95% CI (2.16–7.61); IC = 1.8; 95% CI (0.72–2.52)] was induced by moxifloxacin. Psychiatric AEs, including suicide [*n* = 15; ROR = 18.87; 95% CI (11.12–32.04); IC = 3.51; 95% CI (2.63–4.11)]and attempted suicide [*n* = 8; ROR = 5.07; 95% CI (2.5–10.3); IC = 1.99; 95% CI (0.78–2.8)], were examined for ozenoxacin. Ozenoxacin was also associated with other AEs, including overdose [*n* = 21; ROR = 4.98; 95% CI (3.16–7.85); IC = 2.07; 95% CI (1.33–2.58)], fetal exposure during pregnancy [*n* = 20; ROR = 4.58; 95% CI (2.88–7.28); IC = 1.96; 95% CI (1.21–2.48)], seizure [*n* = 16; ROR = 6.01; 95% CI (3.6–10.04); IC = 2.29; 95% CI (1.45–2.87)] and so on (Table 1). The signal strengths were calculated in the 12–18-year age group (See Supplementary Table S1).

**Table 2:** Signal strength for the top 20 most frequently AEs with quinolones at PT level in FAERS.

Drugs	SOC	PT	N	ROR	95%CI		IC	95%CI	
					Lower limit	Upper limit		Lower limit	Upper limit
Ciprofloxacin	General Disorders and Administration Site Conditions	Pyrexia	236	2.39	2.09	2.74	1.17	0.95	1.33
	Gastrointestinal Disorders	Vomiting	169	1.55	1.32	1.81	0.59	0.34	0.77
	Gastrointestinal Disorders	Diarrhoea	126	2.52	2.1	3.01	1.27	0.98	1.48
	General Disorders and Administration Site Conditions	Drug Interaction	117	4.96	4.11	5.98	2.21	1.9	2.43
	Gastrointestinal Disorders	Abdominal Pain	96	2.2	1.8	2.71	1.09	0.76	1.34
	Respiratory, Thoracic and Mediastinal Disorders	Dyspnoea	96	2.01	1.63	2.46	0.96	0.63	1.21
	Gastrointestinal Disorders	Nausea	94	1.53	1.24	1.88	0.58	0.24	0.83
	General Disorders and Administration Site Conditions	Pain	79	2.44	1.95	3.06	1.24	0.87	1.51
	Blood And Lymphatic System Disorders	Febrile Neutropenia	77	3.54	2.82	4.45	1.75	1.38	2.03
	Infections And Infestations	Pneumonia	73	2.07	1.64	2.62	1.01	0.62	1.29
	Infections And Infestations	Sepsis	73	4.17	3.3	5.28	1.98	1.59	2.26
	Blood And Lymphatic System Disorders	Neutropenia	70	2.99	2.35	3.8	1.52	1.12	1.81
	General Disorders and Administration Site Conditions	Condition Aggravated	69	1.65	1.29	2.09	0.69	0.29	0.98
	General Disorders and Administration Site Conditions	Malaise	60	1.79	1.38	2.31	0.81	0.38	1.11
	Renal And Urinary Disorders	Acute Kidney Injury	59	4.36	3.36	5.65	2.03	1.6	2.35
	Vascular Disorders	Hypotension	59	1.92	1.48	2.48	0.91	0.47	1.22
	General Disorders and Administration Site Conditions	Mucosal Inflammation	57	5.88	4.5	7.67	2.44	2	2.75
	Blood And Lymphatic System Disorders	Thrombocytopenia	55	3.06	2.34	4.01	1.55	1.11	1.88
	Musculoskeletal And Connective Tissue Disorders	Arthralgia	51	2.25	1.7	2.97	1.13	0.66	1.46
	Gastrointestinal Disorders	Crohn’s Disease	49	2.46	1.85	3.27	1.25	0.78	1.59
Levofloxacin	Blood And Lymphatic System Disorders	Febrile Neutropenia	120	13.74	11.35	16.64	3.54	3.23	3.75
	General Disorders and Administration Site Conditions	Pyrexia	103	2.36	1.93	2.89	1.15	0.83	1.39
	Gastrointestinal Disorders	Vomiting	89	1.87	1.51	2.32	0.84	0.49	1.1
	Vascular Disorders	Hypotension	61	4.66	3.6	6.03	2.11	1.68	2.41
	Musculoskeletal And Connective Tissue Disorders	Arthralgia	58	6.02	4.62	7.85	2.46	2.02	2.77
	Infections And Infestations	Pneumonia	55	3.62	2.76	4.75	1.77	1.32	2.09
	Metabolism And Nutrition Disorders	Hypokalaemia	46	13.83	10.25	18.65	3.52	3.03	3.87
	Gastrointestinal Disorders	Diarrhoea	45	2.01	1.49	2.71	0.96	0.47	1.32
	Blood And Lymphatic System Disorders	Neutropenia	44	4.31	3.18	5.83	2	1.5	2.36
	Blood And Lymphatic System Disorders	Aplastic Anaemia	43	40.62	29.56	55.81	4.68	4.17	5.04
	Gastrointestinal Disorders	Nausea	42	1.55	1.14	2.11	0.6	0.09	0.97
	Respiratory, Thoracic and Mediastinal Disorders	Dyspnoea	40	1.89	1.38	2.59	0.88	0.35	1.25
	General Disorders and Administration Site Conditions	Mucosal Inflammation	38	8.93	6.45	12.38	2.96	2.42	3.34
	Infections And Infestations	Infective Pulmonary Exacerbation of Cystic Fibrosis	36	15.38	10.97	21.55	3.61	3.06	4.01
	Gastrointestinal Disorders	Abdominal Pain	35	1.81	1.29	2.53	0.82	0.25	1.22
	Respiratory, Thoracic and Mediastinal Disorders	Cough	34	2.08	1.48	2.93	1.01	0.44	1.42
	Investigations	Alanine Aminotransferase Increased	31	4.16	2.91	5.95	1.95	1.35	2.37
	Investigations	Aspartate Aminotransferase Increased	31	4.58	3.2	6.55	2.08	1.48	2.5
	Metabolism And Nutrition Disorders	Decreased Appetite	30	2.46	1.71	3.54	1.24	0.63	1.67
	Blood And Lymphatic System Disorders	Thrombocytopenia	29	3.67	2.53	5.31	1.78	1.16	2.22
Moxifloxacin	Injury, Poisoning and Procedural Complications	Off Label Use	48	2.07	1.53	2.81	0.94	0.46	1.28
	Gastrointestinal Disorders	Vomiting	28	1.87	1.27	2.75	0.83	0.2	1.28
	Blood And Lymphatic System Disorders	Neutropenia	18	5.64	3.51	9.06	2.27	1.48	2.82
	Injury, Poisoning and Procedural Complications	Product Use Issue	18	1.9	1.18	3.05	0.86	0.06	1.41
	General Disorders and Administration Site Conditions	Drug Interaction	13	3.87	2.22	6.73	1.77	0.83	2.41
	Eye Disorders	Ocular Hyperaemia	12	15.44	8.66	27.53	3.25	2.28	3.92
	Nervous System Disorders	Dyskinesia	11	7.01	3.84	12.79	2.44	1.42	3.14
	Respiratory, Thoracic and Mediastinal Disorders	Respiratory Arrest	11	8.63	4.73	15.74	2.66	1.64	3.36
	Cardiac Disorders	Cardiac Arrest	10	4.06	2.16	7.61	1.8	0.72	2.52
	Gastrointestinal Disorders	Abdominal Distension	10	8.29	4.42	15.57	2.59	1.51	3.32
	Gastrointestinal Disorders	Pancreatitis	10	9.43	5.02	17.71	2.72	1.64	3.45
	General Disorders and Administration Site Conditions	Drug Resistance	10	12.6	6.7	23.68	2.99	1.91	3.72
	General Disorders and Administration Site Conditions	Mucosal Inflammation	10	7.33	3.9	13.76	2.46	1.39	3.19
	General Disorders and Administration Site Conditions	Oedema Peripheral	10	9.36	4.98	17.57	2.71	1.64	3.44
	Immune System Disorders	Anaphylactic Reaction	10	5.04	2.69	9.46	2.05	0.97	2.78
	Infections And Infestations	Herpes Zoster Meningoencephalitis	10	692.51	321.92	1489.7	4.33	3.25	5.06
	Investigations	Alanine Aminotransferase Increased	10	4.24	2.26	7.96	1.85	0.77	2.58
	Investigations	Aspartate Aminotransferase Increased	9	4.19	2.16	8.12	1.82	0.68	2.58
	Blood And Lymphatic System Disorders	Aplastic Anaemia	8	22.02	10.87	44.6	3.28	2.06	4.08
	Metabolism And Nutrition Disorders	Lactic Acidosis	8	12.44	6.16	25.15	2.87	1.66	3.67
Ofloxacin	General Disorders and Administration Site Conditions	Drug Resistance	18	36.74	22.65	59.59	4.16	3.37	4.71
	Nervous System Disorders	Neuropathy Peripheral	10	27.02	14.3	51.08	3.56	2.48	4.29
	Blood And Lymphatic System Disorders	Anaemia	9	5.59	2.87	10.88	2.13	0.99	2.9
	Infections And Infestations	Infection	9	6.97	3.58	13.58	2.37	1.23	3.13
	Investigations	Alanine Aminotransferase Increased	7	4.55	2.15	9.66	1.85	0.55	2.71
	Respiratory, Thoracic and Mediastinal Disorders	Respiratory Failure	7	3.97	1.87	8.43	1.7	0.4	2.56
	Eye Disorders	Eye Pain	6	17.18	7.62	38.74	2.92	1.5	3.83
	Infections And Infestations	Upper Respiratory Tract Infection	6	7.73	3.43	17.39	2.33	0.91	3.24
	Infections And Infestations	Staphylococcal Infection	6	9.58	4.26	21.59	2.51	1.09	3.42
	Injury, Poisoning and Procedural Complications	Drug Dispensing Error	6	14.25	6.32	32.13	2.8	1.38	3.71
	Nervous System Disorders	Hydrocephalus	6	17.88	7.93	40.33	2.94	1.53	3.85
	Respiratory, Thoracic and Mediastinal Disorders	Asthma	6	4.32	1.92	9.72	1.76	0.35	2.67
	Skin And Subcutaneous Tissue Disorders	Stevens-Johnson Syndrome	6	6.53	2.9	14.7	2.17	0.76	3.08
	Skin And Subcutaneous Tissue Disorders	Drug Rash with Eosinophilia and Systemic Symptoms	6	36.77	16.24	83.24	3.28	1.86	4.19
	Eye Disorders	Ocular Hyperaemia	5	9.67	3.98	23.49	2.42	0.86	3.4
	General Disorders and Administration Site Conditions	Disease Progression	5	5.84	2.4	14.16	2	0.44	2.99
	Immune System Disorders	Immune Reconstitution Inflammatory Syndrome	5	61.59	25.08	151.21	3.23	1.67	4.22
	Infections And Infestations	Urinary Tract Infection	5	6.97	2.87	16.91	2.16	0.6	3.14
	Infections And Infestations	Tuberculoma Of Central Nervous System	5	319.22	123.57	824.66	3.41	1.85	4.39
	Infections And Infestations	Tuberculosis	5	58.18	23.71	142.76	3.22	1.66	4.21
Ozenoxacin	Cardiac Disorders	Cardiac Arrest	22	19.83	12.69	30.98	3.68	2.96	4.18
	Injury, Poisoning and Procedural Complications	Overdose	21	4.98	3.16	7.85	2.07	1.33	2.58
	Injury, Poisoning and Procedural Complications	Foetal Exposure During Pregnancy	20	4.58	2.88	7.28	1.96	1.21	2.48
	Injury, Poisoning and Procedural Complications	Toxicity to Various Agents	20	7.5	4.72	11.94	2.57	1.82	3.1
	Nervous System Disorders	Seizure	16	6.01	3.6	10.04	2.29	1.45	2.87
	Psychiatric Disorders	Completed Suicide	15	18.87	11.12	32.04	3.51	2.63	4.11
	Cardiac Disorders	Brugada Syndrome	13	699.65	375.06	1305.14	4.69	3.75	5.33
	Nervous System Disorders	Coma	12	16.45	9.15	29.56	3.29	2.31	3.95
	Nervous System Disorders	Somnolence	12	3.77	2.1	6.77	1.7	0.72	2.37
	Cardiac Disorders	Cardio-Respiratory Arrest	10	14.92	7.88	28.26	3.12	2.04	3.84
	Skin And Subcutaneous Tissue Disorders	Urticaria	10	3.89	2.06	7.35	1.72	0.65	2.45
	Cardiac Disorders	Tachycardia	9	4.72	2.42	9.23	1.94	0.8	2.7
	Nervous System Disorders	Status Epilepticus	9	16.42	8.39	32.13	3.14	2	3.9
	Pregnancy, Puerperium and Perinatal Conditions	Small for Dates Baby	9	14.7	7.51	28.77	3.05	1.92	3.82
	Psychiatric Disorders	Suicide Attempt	8	5.07	2.5	10.3	1.99	0.78	2.8
	Skin And Subcutaneous Tissue Disorders	Dermatitis Atopic	8	24.44	12	49.78	3.33	2.12	4.13
	Vascular Disorders	Hypotension	8	3.83	1.88	7.78	1.68	0.46	2.48
	Investigations	Electrocardiogram Qrs Complex Prolonged	7	47.13	22.01	100.88	3.51	2.21	4.37
	Respiratory, Thoracic and Mediastinal Disorders	Asthma	7	6.69	3.14	14.26	2.24	0.94	3.1
	Respiratory, Thoracic and Mediastinal Disorders	Neonatal Respiratory Distress Syndrome	7	16.06	7.53	34.23	2.97	1.67	3.83

## Discussion

The ROR and BCPNN methods were used to assess the association between AEs and quinolone treatment in pediatrics department and further to evaluate its medicine safety in children. Our findings were generally consistent with the AEs in the manufacturer’s instructions, demonstrating the validity of the data mining methods.

Our study found that the most prevalent AEs associated with quinolone use (oral or intravenous administration) in children were gastrointestinal reactions. We found three significant (ciprofloxacin, levofloxacin, and moxifloxacin) signals for vomiting, and two significant signals (ciprofloxacin and levofloxacin) for nausea, abdominal pain, and diarrhoea. Our analysis found a significant signal for abdominal distension with moxifloxacin, which were consistent with a meta-analysis by Li et al. (13) and a similar studies in adults (24, 25). A strong signal of arthralgia was elicited from ciprofloxacin and levofloxacin, which were similar to the reported research by Adefurin et al. (26).

In terms of age, 12–18-year-old patients were the most reported age group in our study, which might be related to the higher frequency of quinolone use in this population (13). FQ-induced tendon disorders were reported to occur tendon ruptures within 1 week of administration and tendinopathies within the first month (27), Similarly, we observed that the onset of the most frequent FQ-musculoskeletal ADRs was 7 days for the median treatment with ciprofloxacin, 8 days for levofloxacin, 5 days for moxifloxacin and ofloxacin, and 41 days for ozenoxacin. Peripheral neuropathy caused by quinolones in adults has been reported in previous studies (28), but has not been observed in children., two signals of CNS disorders, dyskinesia (ROR = 7.01, IC = 2.44) and peripheral neuropathy (ROR = 27.02, IC = 3.56) were detected in this study, which require further study.

Our analysis found significant signals of ciprofloxacin and levofloxacin in musculoskeletal adverse events, which is showed in the instructions. An estimated risk of one musculoskeletal AE for every 62.5 patients and a 57% increased risk of arthropathy associated with ciprofloxacin was reported by Adefurin et al. (26). Our results identified that musculoskeletal pain were associated with levofloxacin and ciprofloxacin, which are concordant with the previous findings (2, 29). Although quinolone use in children may be lead to musculoskeletal AEs, long-term follow-up studies suggest that they are reversible and do not contribute to growth problems (12). The exact pathophysiology of tendon injury induced by FQ is still mysterious (27). FQ-induced tendon damage is thought to be associated with oxidative stress and mitochondrial toxicity (28). FQs interact with regulatory proteins of the tenant and further damage the tendon structure. Besides, the final event in the pathogenetic mechanism has been suggested to be apoptosis (30).

Cardiac arrest was uncommon, only linked to moxifloxacin and ozenoxacin. An RCT conducted by Stefan et al. (2) found a drug-related QT prolongation of 9.3% and a recent systematic review and meta-analysis identified a prolonged electrocardiogram QT interval of 0.03% (13). It might be a fatal arrhythmia. Prolongation of the QTc interval increases the risk of Torsade de Pointes (TdP) arrhythmia, especially when the QTc interval exceeds 500 ms or the extension is larger than 60 ms compared to the pre-processing value. There are many risk factors of prolonged electrocardiogram QT interval, including female sex, bradycardia, hypokalemia, hypocalcemia, hypomagnesemia, history of cardiac disease and treatment with more than one QTc-prolonging medication. Therefore, any link between moxifloxacin-induced QTc extension and risk factors was hardly established (2).

For drug resistance, our findings showed significant signals in moxifloxacin and ofloxacin.

Therefore, the occurrence of drug resistance should also be closely monitored in children. The use of FQs elevates in this population, and drug resistance will also consequentially increase. One study reported (31) the correlation between FQs use and the emergence of ciprofloxacin and levofloxacin resistance in gram-negative bacilli in hospitalized children.The current study identified drug resistance signals induced by moxifloxacin and ofloxacin.

Quinolones have also been associated with seizures in children. We only found significant signals in ozenoxacin. An increase in seizures (0.63%) was found among children without CNS disorders (0.02%) after using quinolones such as ciprofloxacin, levofloxacin, and gatifloxacin (32). Ozenoxacin is a novel, non-fluoroquinated quinolone, and approved for topical use by the FDA in 2017. This drug is bactericidal against gram-positive pathogens, including methicillin-resistant and methicillin-sensitive *Staphylococcus aureus*. Ozenoxacin lacks a fluorine atom and have fewer side effects than other fluoroquinolones (33). Ofloxacin produced typical quinolone-induced lesions in the articular cartilage of three of 10 juvenile rats, while chondrotoxicity was unassociated with ozenoxacin (34). Oral administration of ozenoxacin in juvenile dogs demonstrated no chondrotoxicity or toxicologically in select target organs. Ozenoxacin-associated rhinitis, and rare AEs, such as cardiac arrest and epilepsy, were identified by Savion Gropper et al. (35). Only rosacea and seborrheic dermatitis are listed as AEs on the ozenoxacin package insert, while the current study identified strong signals for coma, somnolence, suicide and attempted suicide. Although the number of AEs was low, these side effects should be closely monitored during ozenoxacin use in children. Further studies are needed to assess its safety profile in this population.

Our study has several limitations. First, AEs are often underreported in spontaneous reporting systems, which is caused by various factors, such as the underreporting, absence, and exclusion of healthy individuals, the lack of a denominator, and the presence of potential confounders (36). The FAERS database is not appropriate for estimating incidence rates, due to the absence of a denominator (37). Second, most reporters are from the United States, European and South American countries; therefore, it is uncertain whether the findings are applicable to individuals of other races and ethnicities. Third, although most of the reports (75.4%) are reported by health professionals (physician, pharmacist and other health-professional), and the rest of the cases submitted by consumers have not been verified, and the reliability of the reported information may be affected by these reasons. Last, the FDA does not require a proof of the cause-and-effect relationship between AE and drug, so the reports often lack detailed information about the AE.

## Conclusion

In summary, this study provides an objective reference for pharmacovigilance work by mining the safety signals of quinolone use in children. Focus should be placed on AEs with strong real-world signals, such as the cardiac arrest and suicide death associated with ozefloxacin that are not included in the label. In addition, both the risk and benefit should be appropriately weighed when quinolones are prescribed for children. Pharmaceutical care should be strengthened for patients with risk factors for heart disease, skeletal muscle disease, and hematological conditions. Close attention should be paid to disease progression and timely intervention measures should be taken when AEs occur to reduce the risk of poor outcomes.

## Data Availability

Publicly available datasets were analyzed in this study. This data can be found here: https://www.fda.gov/drugs/questions-and-answers-fdas-adverse-event-reporting-system-faers/fda-adverse-event-reporting-system-faers-latest-quarterly-data-files.
