# Supplementary material for: Disproportionality analysis of quinolone safety in children using data from the FDA adverse event reporting system (FAERS)
Source: Front Pediatr. 2023 Jan 11;10:1069504. doi: 10.3389/fped.2022.1069504 (PMC9874243; doi:10.3389/fped.2022.1069504)
Supplement: Supplementary file 1 [file Datasheet1.docx]

Supplementary formula

ROR method:

$ROR=\frac{a/c}{b/d}$ ^^

Bayesian confidence propagation network algorithm:

$IC=\text{log}_{2}\frac{a+0.5}{a_{\exp}+0.5}$ $a_{\exp}=\frac{(a+b)*(a+c)}{(a+b+c+d)}$

$$IC_{025}=\text{IC-3.3*}\left(\alpha+0.5\right)^{-0.5}-2*\left(\alpha+0.5\right)^{-1.5}$$

$$IC_{975}=\text{IC}+\text{2.4*}\left(\alpha+0.5\right)^{-0.5}-0.5*\left(\alpha+0.5\right)^{-1.5}$$

**α** is the number of AE cases attributable to the suspected drug; **b** is the number of AE cases attributable to other drugs; **c** is the number of other AE cases attributable to the suspected drug; **d** is the number of other AE cases attributable to other drugs.

Supplementary Table S1. Signal strength for the top 20 most frequently AEs with quinolones at PT level in FAERS in 12- to 18-year-old group

| **Drugs** | **SOC** | **PT** | **N** | **ROR** | **95%CI** | | **IC** | **95%CI** | |
| --- | --- | --- | --- | --- | --- | --- | --- | --- | --- |
|  |  |  |  |  | **Lower limit** | **Upper limit** |  | **Lower limit** | **Upper limit** |
| Ciprofloxacin | General disorders and administration site conditions | Pyrexia | 91 | 2.74 | 2.21 | 3.40 | 1.37 | 1.02 | 1.62 |
|  | Gastrointestinal disorders | Vomiting | 85 | 1.94 | 1.56 | 2.42 | 0.90 | 0.54 | 1.16 |
|  | Gastrointestinal disorders | Nausea | 70 | 1.73 | 1.36 | 2.21 | 0.75 | 0.35 | 1.03 |
|  | General disorders and administration site conditions | Pain | 54 | 2.80 | 2.13 | 3.68 | 1.41 | 0.96 | 1.73 |
|  | Gastrointestinal disorders | Abdominal pain | 49 | 1.94 | 1.45 | 2.58 | 0.91 | 0.43 | 1.25 |
|  | Respiratory, thoracic and mediastinal disorders | Dyspnoea | 47 | 2.03 | 1.52 | 2.72 | 0.97 | 0.49 | 1.32 |
|  | Gastrointestinal disorders | Diarrhoea | 44 | 2.25 | 1.66 | 3.05 | 1.11 | 0.61 | 1.47 |
|  | Gastrointestinal disorders | Crohn's disease | 41 | 2.34 | 1.71 | 3.21 | 1.17 | 0.65 | 1.54 |
|  | Renal and urinary disorders | Acute kidney injury | 41 | 5.54 | 4.04 | 7.60 | 2.33 | 1.81 | 2.70 |
|  | General disorders and administration site conditions | Drug interaction | 39 | 3.25 | 2.35 | 4.48 | 1.61 | 1.08 | 1.99 |
|  | Blood and lymphatic system disorders | Febrile neutropenia | 38 | 5.06 | 3.65 | 7.02 | 2.20 | 1.66 | 2.59 |
|  | Blood and lymphatic system disorders | Neutropenia | 36 | 3.88 | 2.78 | 5.42 | 1.85 | 1.30 | 2.25 |
|  | Infections and infestations | Infective pulmonary Exacerbation of cystic fibrosis | 36 | 10.35 | 7.36 | 14.54 | 3.10 | 2.55 | 3.50 |
|  | Respiratory, thoracic and mediastinal disorders | Cough | 34 | 2.72 | 1.93 | 3.84 | 1.37 | 0.80 | 1.78 |
|  | Musculoskeletal and connective tissue disorders | Arthralgia | 33 | 2.21 | 1.56 | 3.13 | 1.09 | 0.51 | 1.50 |
|  | Blood and lymphatic system disorders | Thrombocytopenia | 32 | 4.75 | 3.33 | 6.77 | 2.11 | 1.52 | 2.53 |
|  | General disorders and administration site conditions | Mucosal inflammation | 30 | 8.32 | 5.74 | 12.04 | 2.82 | 2.21 | 3.25 |
|  | Investigations | Haemoglobin decreased | 29 | 6.55 | 4.51 | 9.53 | 2.52 | 1.90 | 2.96 |
|  | Infections and infestations | Pneumonia | 27 | 2.62 | 1.79 | 3.85 | 1.32 | 0.68 | 1.77 |
|  | Renal and urinary disorders | Renal failure acute | 26 | 6.64 | 4.47 | 9.85 | 2.52 | 1.87 | 2.99 |
| Levofloxacin | Gastrointestinal disorders | Vomiting | 43 | 2.23 | 1.64 | 3.05 | 1.08 | 0.57 | 1.44 |
|  | General disorders and administration site conditions | Pyrexia | 43 | 2.92 | 2.14 | 3.99 | 1.44 | 0.93 | 1.80 |
|  | Blood and lymphatic system disorders | Febrile neutropenia | 37 | 11.56 | 8.23 | 16.19 | 3.234 | 2.69 | 3.62 |
|  | Musculoskeletal and connective tissue disorders | Arthralgia | 37 | 5.85 | 4.18 | 8.17 | 2.37 | 1.82 | 2.76 |
|  | Vascular disorders | Hypotension | 36 | 5.11 | 3.64 | 7.18 | 2.19 | 1.64 | 2.59 |
|  | Infections and infestations | Pneumonia | 31 | 7.06 | 4.91 | 10.17 | 2.61 | 2.01 | 3.03 |
|  | Metabolism and nutrition disorders | Hypokalaemia | 30 | 20.62 | 14.16 | 30.03 | 3.86 | 3.25 | 4.29 |
|  | Gastrointestinal disorders | Abdominal pain | 26 | 2.33 | 1.57 | 3.46 | 1.14 | 0.49 | 1.61 |
|  | Respiratory, thoracic and mediastinal disorders | Dyspnoea | 24 | 2.35 | 1.56 | 3.53 | 1.15 | 0.47 | 1.63 |
|  | Infections and infestations | Infective pulmonary Exacerbation of cystic fibrosis | 23 | 14.83 | 9.70 | 22.67 | 3.44 | 2.74 | 3.93 |
|  | Blood and lymphatic system disorders | Neutropenia | 22 | 5.38 | 3.50 | 8.25 | 2.24 | 1.52 | 2.74 |
|  | General disorders and administration site conditions | Asthenia | 22 | 4.49 | 2.93 | 6.90 | 2.01 | 1.30 | 2.51 |
|  | General disorders and administration site conditions | Fatigue | 21 | 1.72 | 1.11 | 2.66 | 0.73 | 0.0013 | 1.25 |
|  | Gastrointestinal disorders | Diarrhoea | 21 | 2.42 | 1.56 | 3.75 | 1.19 | 0.46 | 1.71 |
|  | Respiratory, thoracic and mediastinal disorders | Cough | 21 | 3.82 | 2.47 | 5.92 | 1.80 | 1.066 | 2.31 |
|  | Injury, poisoning and procedural complications | Toxicity to various agents | 20 | 1.88 | 1.20 | 2.94 | 0.85 | 0.10 | 1.38 |
|  | Nervous system disorders | Somnolence | 20 | 2.09 | 1.34 | 3.27 | 0.99 | 0.25 | 1.52 |
|  | Metabolism and nutrition disorders | Decreased appetite | 19 | 3.75 | 2.37 | 5.94 | 1.77 | 0.99 | 2.31 |
|  | Nervous system disorders | Neuropathy peripheral | 18 | 17.30 | 10.72 | 27.92 | 3.52 | 2.73 | 4.07 |
|  | Investigations | Aspartate aminotransferase increased | 17 | 6.44 | 3.96 | 10.47 | 2.43 | 1.62 | 2.10 |
| Moxifloxacin | Immune system disorders | Anaphylactic reaction | 9 | 9.90 | 5.05 | 19.41 | 2.70 | 1.56 | 3.46 |
|  | Investigations | Alanine aminotransferase increased | 8 | 9.16 | 4.49 | 18.67 | 2.59 | 1.37 | 3.39 |
|  | Skin and subcutaneous tissue disorders | Urticaria | 8 | 3.28 | 1.61 | 6.68 | 1.49 | 0.28 | 2.29 |
|  | General disorders and administration site conditions | Drug resistance | 7 | 30.02 | 13.98 | 64.45 | 3.33 | 2.02 | 4.18 |
|  | Investigations | Aspartate aminotransferase increased | 6 | 7.62 | 3.37 | 17.25 | 2.30 | 0.89 | 3.22 |
|  | Gastrointestinal disorders | Gastrointestinal disorder | 6 | 13.55 | 5.98 | 30.73 | 2.76 | 1.34 | 3.67 |
|  | Metabolism and nutrition disorders | Hypoglycaemia | 5 | 12.09 | 4.95 | 29.54 | 2.57 | 1.00 | 3.55 |
|  | General disorders and administration site conditions | Hypothermia | 5 | 25.45 | 10.37 | 62.45 | 2.96 | 1.40 | 3.95 |
|  | Hepatobiliary disorders | Hepatitis | 5 | 16.11 | 6.59 | 39.42 | 2.74 | 1.18 | 3.73 |
|  | Endocrine disorders | Hypothyroidism | 5 | 23.96 | 9.77 | 58.76 | 2.94 | 1.37 | 3.92 |
|  | Infections and infestations | Tuberculoma of central nervous system | 5 | 695.70 | 235.25 | 2057.37 | 3.43 | 1.87 | 4.41 |
|  | Vascular disorders | Flushing | 5 | 7.84 | 3.21 | 19.13 | 2.25 | 0.69 | 3.23 |
|  | Ear and labyrinth disorders | Hypoacusis | 5 | 70.96 | 28.52 | 176.54 | 3.25 | 1.69 | 4.24 |
|  | Blood and lymphatic system disorders | Bone marrow toxicity | 5 | 1739.29 | 462.96 | 6534.29 | 3.44 | 1.88 | 4.42 |
|  | Infections and infestations | Conjunctivitis | 4 | 17.94 | 6.61 | 48.66 | 2.62 | 0.86 | 3.70 |
|  | Ear and labyrinth disorders | Deafness bilateral | 4 | 138.29 | 48.93 | 390.85 | 3.08 | 1.31 | 4.16 |
|  | Vascular disorders | Circulatory collapse | 3 | 13.20 | 4.19 | 41.57 | 2.26 | 0.19 | 3.46 |
|  | Infections and infestations | Disseminated tuberculosis | 3 | 108.53 | 33.18 | 354.99 | 2.72 | 0.65 | 3.93 |
|  | Gastrointestinal disorders | Gastritis | 3 | 11.50 | 3.66 | 36.20 | 2.19 | 0.12 | 3.40 |
|  | Infections and infestations | Pulmonary tuberculosis | 3 | 67.60 | 21.00 | 217.57 | 2.68 | 0.61 | 3.88 |
| Ofloxacin | Gastrointestinal disorders | Vomiting | 8 | 2.90 | 1.40 | 6.02 | 1.30 | 0.092 | 2.11 |
|  | Nervous system disorders | Neuropathy peripheral | 6 | 40.14 | 17.40 | 92.56 | 3.29 | 1.88 | 4.20 |
|  | General disorders and administration site conditions | Drug resistance | 6 | 53.95 | 23.35 | 124.64 | 3.39 | 1.97 | 4.30 |
|  | Skin and subcutaneous tissue disorders | Stevens-johnson syndrome | 5 | 18.66 | 7.54 | 46.20 | 2.81 | 1.25 | 3.79 |
|  | Infections and infestations | Staphylococcal infection | 4 | 22.04 | 8.04 | 60.39 | 2.70 | 0.94 | 3.78 |
|  | Nervous system disorders | Petit mal epilepsy | 4 | 51.76 | 18.80 | 142.50 | 2.95 | 1.18 | 4.03 |
|  | Eye disorders | Optic neuropathy | 4 | 481.34 | 163.30 | 1418.78 | 3.14 | 1.38 | 4.22 |
|  | Injury, poisoning and procedural complications | Product dose omission issue | 4 | 6.98 | 2.55 | 19.08 | 2.03 | 0.27 | 3.11 |
|  | Infections and infestations | Pneumonia | 4 | 6.12 | 2.24 | 16.73 | 1.93 | 0.16 | 3.01 |
|  | Respiratory, thoracic and mediastinal disorders | Respiratory failure | 4 | 12.05 | 4.40 | 32.95 | 2.41 | 0.64 | 3.49 |
|  | Eye disorders | Eye rolling | 4 | 213.90 | 75.68 | 604.59 | 3.11 | 1.35 | 4.19 |
|  | Immune system disorders | Anaphylactic shock | 4 | 31.01 | 11.30 | 85.08 | 2.82 | 1.06 | 3.90 |
|  | Nervous system disorders | Clonus | 4 | 78.54 | 28.40 | 217.20 | 3.02 | 1.25 | 4.10 |
|  | Immune system disorders | Immune reconstitution inflammatory syndrome | 4 | 222.13 | 78.49 | 628.67 | 3.11 | 1.35 | 4.19 |
|  | Renal and urinary disorders | Urinary incontinence | 4 | 42.58 | 15.49 | 117.06 | 2.91 | 1.14 | 3.99 |
|  | Nervous system disorders | Cerebellar syndrome | 3 | 186.01 | 56.70 | 610.24 | 2.76 | 0.69 | 3.96 |
|  | Nervous system disorders | Intracranial pressure increased | 3 | 22.43 | 7.05 | 71.30 | 2.45 | 0.38 | 3.66 |
|  | Nervous system disorders | Intention tremor | 3 | 450.39 | 130.73 | 1551.71 | 2.78 | 0.72 | 3.99 |
|  | Nervous system disorders | Hydrocephalus | 3 | 82.25 | 25.57 | 264.57 | 2.70 | 0.63 | 3.91 |
|  | Infections and infestations | Hypopyon | 3 | 4279.02 | 705.60 | 25949.70 | 2.80 | 0.73 | 4.01 |
| Ozenoxacin | Injury, poisoning and procedural complications | Overdose | 21 | 6.91 | 4.28 | 11.16 | 2.37 | 1.63 | 2.88 |
|  | Injury, poisoning and procedural complications | Toxicity to various agents | 20 | 12.30 | 7.55 | 20.04 | 3.05 | 2.30 | 3.57 |
|  | Cardiac disorders | Cardiac arrest | 19 | 34.15 | 20.73 | 56.28 | 4.04 | 3.27 | 4.58 |
|  | Psychiatric disorders | Completed suicide | 15 | 17.17 | 9.92 | 29.71 | 3.35 | 2.48 | 3.96 |
|  | Nervous system disorders | Seizure | 14 | 13.08 | 7.44 | 22.99 | 3.07 | 2.17 | 3.69 |
|  | Nervous system disorders | Coma | 12 | 26.90 | 14.70 | 49.22 | 3.63 | 2.66 | 4.30 |
|  | Cardiac disorders | Cardio-respiratory arrest | 10 | 31.60 | 16.41 | 60.87 | 3.62 | 2.54 | 4.35 |
|  | Cardiac disorders | Tachycardia | 9 | 8.49 | 4.28 | 16.84 | 2.53 | 1.39 | 3.29 |
|  | Psychiatric disorders | Suicide attempt | 8 | 4.55 | 2.21 | 9.35 | 1.84 | 0.63 | 2.64 |
|  | Skin and subcutaneous tissue disorders | Urticaria | 7 | 4.78 | 2.22 | 10.29 | 1.87 | 0.57 | 2.72 |
|  | Respiratory, thoracic and mediastinal disorders | Respiratory failure | 6 | 14.63 | 6.40 | 33.44 | 2.80 | 1.38 | 3.71 |
|  | Cardiac disorders | Pulseless electrical activity | 6 | 191.76 | 81.52 | 451.08 | 3.60 | 2.19 | 4.51 |
|  | Renal and urinary disorders | Renal failure | 6 | 14.21 | 6.22 | 32.48 | 2.78 | 1.36 | 3.69 |
|  | Vascular disorders | Hypotension | 6 | 4.60 | 2.02 | 10.50 | 1.80 | 0.38 | 2.71 |
|  | Respiratory, thoracic and mediastinal disorders | Asthma | 6 | 13.04 | 5.71 | 29.79 | 2.72 | 1.30 | 3.63 |
|  | Investigations | Electrocardiogram qrs complex prolonged | 6 | 46.31 | 20.16 | 106.38 | 3.34 | 1.93 | 4.26 |
|  | Metabolism and nutrition disorders | Metabolic acidosis | 5 | 11.84 | 4.81 | 29.12 | 2.54 | 0.98 | 3.53 |
|  | Cardiac disorders | Ventricular dysfunction | 5 | 206.24 | 80.92 | 525.66 | 3.38 | 1.82 | 4.37 |
|  | Renal and urinary disorders | Myoglobinuria | 5 | 275.00 | 106.60 | 709.49 | 3.40 | 1.84 | 4.38 |
|  | Musculoskeletal and connective tissue disorders | Rhabdomyolysis | 5 | 17.32 | 7.03 | 42.67 | 2.77 | 1.21 | 3.76 |


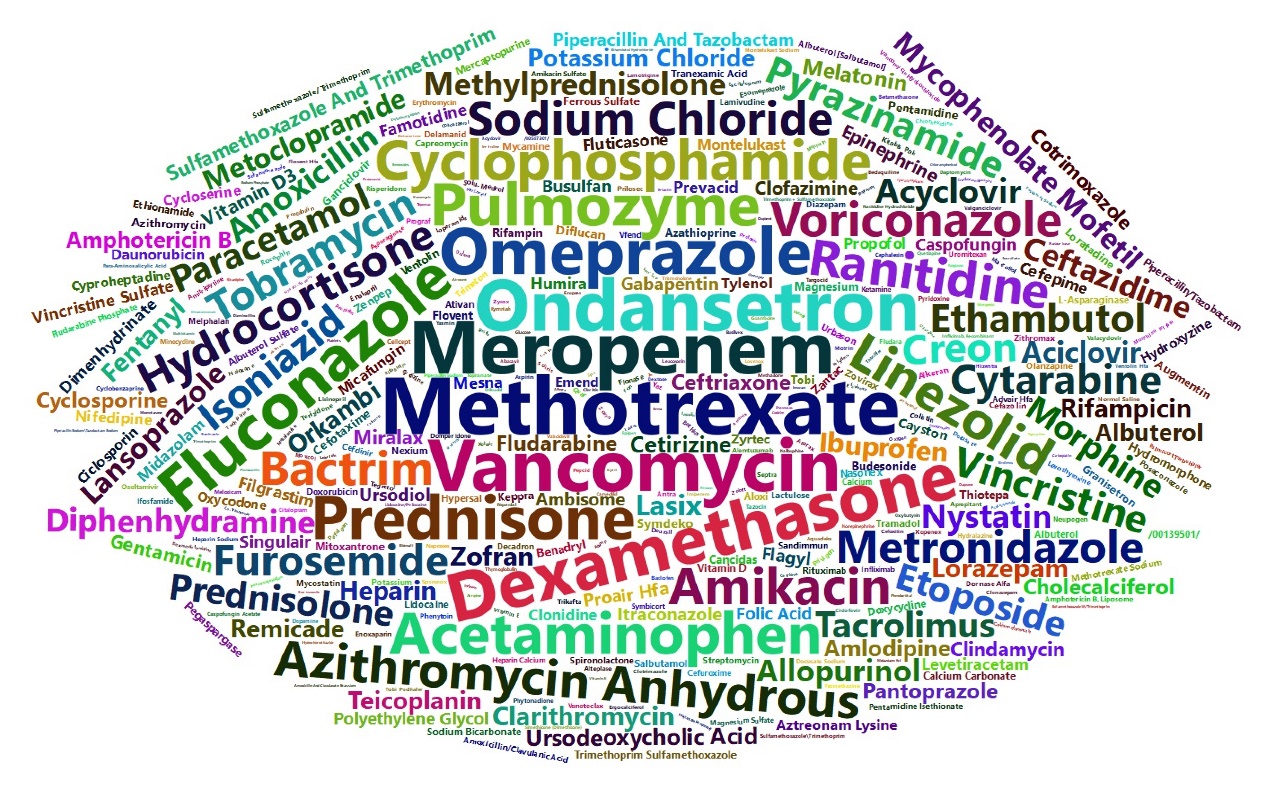


Figure S1. The drugs were concomitant with quinolones. **Note**: The bigger the size of the word, the more it was concomitant use with quinolones.


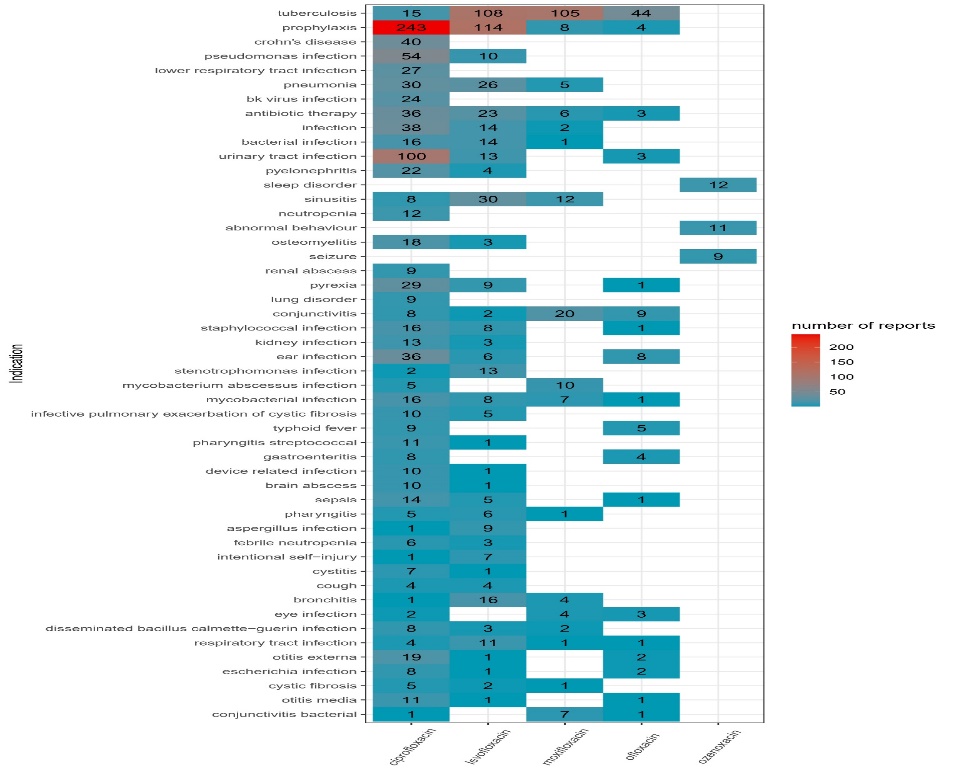


Figure S2. Indications associated with each quinolone involved in top 50 AE reports for Children
